# Primary Surgical Dressings

**Published:** 1889-07

**Authors:** H. McHatton

**Affiliations:** Macon, Ga.


THE ATLANTA MEDICAL AND SURGICAL JOURNAL.

VOL. VI. JULY, 1889. NO. 5.

### Original Communications.

### PRIMARY SURGICAL DRESSINGS.

REPORT ON SURGERY FROM THE SIXTH DISTRICT,

BY H. MCHATTON, M. D.

GENTLEMEN: It is not my intention to go into a discussion on any special surgical subject, but to report some cases that have been of interest to me, and make a few remarks in regard to surgical dressings.

Primary surgical dressings in the present days of antisepsis are of the most vital importance. With the statistics that we have since the introduction of antisepsis, or cleanliness if you prefer, any one that dresses a wound without using the precautions that are recognized by the profession at large is, to say the least, negligent. It is not asking much of a man who does any surgery to keep on hand a case containing carbolic acid, tablets of bichloride, iodoform, a bottle of antisepsic silk, absorbent cotton, bandages, and a fountain syringe. With that he is prepared to dress any wound in an emergency. I do not propose to go into the details of antisepsic drugs and dressings that are found in every text-book and nearly every journal. Bichloride of mercury, carbolic acid and iodoform are those that I use principally. The bichloride is objectionable on account of its corrosive action on instruments. Iodoform is safe, unless it is packed into a cavity or a sinus; in ordinary dressings I have never seen evil results follow its use. A mixture of iodoform and collodion (gr. x to xv to ℥i) makes an excellent dressing for small wounds and plastic operations, when the wound is originally clean, and there is no fear of suppuration. Three or four coats of this are used, and in a few days will peel off, usually leaving a wound that has healed by first intention. In almost all the articles on antiseptic dressings, we are told to leave on our dressings until union has taken place, or we are warned by the temperature that all is not well; this is a good rule in most cases, but like all rules not without exceptions.

The following case occurred in my practice this year:

A. Wilson came under my charge twelve hours after injury, fracture of the first finger, palmar laceration of the second, laceration of the third extending from middle of finger to middle of palm of hand, with fracture of third phalanx; had been dressed with adhesive plaster and numerous sutures applied. Left sutures and cleaned wound as well as possible; dressed, dry iodoform; eleventh day, temperature 98, pulse 74, third finger having lost sensation, determined to remove it; on anæsthesia, I found that all phalanges of second and third fingers were dead, also there was a pus cavity over metacarpal of second, containing about an ounce of pus, and extending nearly to the wrist joint. I also saw in consultation a case of septic poisoning, following traumatic gangrene of the thumb, when the temperature was below a hundred, and the circulation good until the approach of death. In these cases, the rule of temperature would have proven a poor guide; such cases are, of course, rare but it is *well* to be on our guard.

During the year I read an article in some journal on the use of drainage tubes in conjunction with antisepsic dressings, advising the removal of the tube and re-application of the dressing at the end of the first twenty-four or thirty-six hours, as by that time the serous exudation would have ceased, the tube then acting as a foreign body with no compensating benefit. I have tried this plan and can indorse it. It has been my fortune each year to have the after treatment of a large number of surgical cases, where the primary dressing has been applied by others. I find that only in exceptional cases has there been any effort at antisepsis; the wounds are rarely surgically cleaned to start with, and the applications are of the most varied character. Oil of any description seems to be a favorite; turpentine is a good second; turpentine and brown sugar is sometimes seen. My experience is that wounds do not do well under turpentine. I make this statement, knowing it is at variance with the views of my colleagues in this land, where turpentine is used internally, *externally* and eternally.

COURT PLASTER.—When I am called to a case of amputation or extensive laceration, that has been dressed for several days with court plaster, the English language is not sufficiently expressive. Gentleman, if any of you will use it, for the sake of the religion of the man who comes after you, shave off the hair, use the rubber plaster, and leave the cheese-cloth on where it comes in contact with the open wound. Why any man will prefer it to sutures, bandages and absorbent cotton is a *mystery* to me.

SKIN GRAFTING is so much neglected, that I venture to call your attention to the following extract from a paper by myself on ulcers of the lower leg in THE ATLANTA MEDICAL AND SURGICAL JOURNAL of July, 1886:

"Grafting is so simple, so easy and so gratifying in its results, that it astonishes me to see it so seldom used. My method is as follows: As soon as I get the proper granulations (small and velvet-like, of a pinkish color, secreting laudable pus), I furnish myself with the following instruments: a pair of ciliary forceps, (any dressing forceps will do), a pair of scissors and a rubber condom; the latter I cut up in strips about half an inch wide. Take the forceps and pick up as small a piece of skin as you can; snip this off with the scissors as near the forceps as possible; then place it on the granulation. I usually place them in lines, the grafts about a quarter of an inch apart, and the lines about half an inch. Over each of these lines place a piece of the rubber, draw it until it makes pretty firm pressure, and fasten the ends to the sound skin with a piece of adhesive plaster, or by moistening it with chloroform; over all place some absorbent cotton and then your bandage; in seven or eight days you can remove the rubber, and will find from seventy to ninety per cent. of the grafts have taken. In taking your grafts in the manner indicated, you will rarely draw blood, and as there is little or no pain connected with removing them, you will find no trouble in getting volunteers that will give you any reasonable number. I often take them from the patient. In fact, it is much easier to graft an ulcer than to apply a well-fitting bandage to it.

The only deaths that I have had in my surgical practice this *year* have been from tetanus. Harry, a negro of fine physique and in the prime of life, had the ends of all the toes of one foot crushed on April 24th. Antiseptic dressing. 25th, considerable œdema of foot, pulse and temperature normal; May 2d, wound healed; out of doors, 6th; called in haste and found him having tetanic convulsions every five minutes; pulse 150; bromide of soda, ʒi, chloral gr. xv. q. 3. h.

7th, a. m., pulse 100; convulsions every hour.

8th and 9th, no change.

10th, pulse 150; chloral, gr. xx, bromide, gr. xxx, nitro-glycerine, gr. $\frac{1}{50}$, q. 3. h.

11th, pulse 110; convulsions about every two hours; gets considerable sleep.

16th, continued about the same until to-day, pulse went up to 150; *died* in a convulsion.

Simon, of same type and age, was injured on May 8th; the thumb and two fingers were amputated that night. Came under my care on the 9th.

16th, flaps of thumb and second finger have sluffed, bone bare.

17th, amputated. 23d, re-amputated fingers doing well, the other still discharging; pulse 89; temperature 99. 24th, tetanus set in suddenly this a. m.; nitro-glycerine, $\frac{1}{50}$; chloral, gr. xxx, q. 3. h. Opened all wounds thoroughly. 25th, died at 10 a. m.

In each of these cases there had been a succession of cold, wet days previous to the seizure. They were both out of doors and exposed to the weather; we all know the etiological relation that cold and dampness bear to tetanus. I even believe that the prevalence of the disease in the negro may be to a great extent dependent on their surroundings.

These cases were seen in consultation by Dr. H. J. Williams. Amputation, nerve stretching, and cutting were discussed; but as there was no aura, no tenderness on trunk of nerve, and the seizures did not begin in the injured limb, they did not seem to be cases for surgical treatment.

From the effect that the nitro-glycerine had on the first case, I think it well worthy of a more extended trial in tetanus.

A case of septicæmia following amputation is probably worthy of consideration, on account of the unusual temperature curve. You will see by the chart that progress was good until the 16th day, when the temperature went at one jump from 99.8 to 105, pulse from 98 to 120, gradually falling again until the 19th day, when they were respectively 84 and 99½; on the 22d day, 99 and 90, running up to 104 and 120 on the 23d, and 106 and 130 on the 24th; on the 29th, 84 and 98½. Convalescence being from that time uninterrupted.

This is the only case of sepsis that I have had following one of my own operations in the past six years. The hygienic surroundings were such that antisepsis could not be carried out as it should have been; as the patient was in a malarial region, the question of malarial infection of course came up, but the diagnostic symptoms of sepsis were not to be mistaken. My opinion, as is also that of Dr. Williams who saw the case, is that this patient had two distinct septic infections. The treatment consisted in keeping the wound as clean as possible. Quinine, iron, opium, stimulants and nourishment.

HYDROPHOBIA.—In May of last year I had a so-called case of this disease, that in my estimation is a fair type of the majority of the cases that run through the daily papers in a sensational manner, as this one did. A planter of this city requested me to go to his place to see the case, saying that it had been so diagnosed by his neighboring physicians and a fatal prognosis given. I being unable to attend requested him to have the case brought to this city, and I would bear all expenses if it proved to be hydrophobia. Several days afterwards I heard a commotion at my office door, and was surprised to see three negro men bringing a small boy with his hands tied up in bags. This proved to be my case of hydrophobia, with the following history:

Negro boy, 11 years old, while kicking at a dog had his bare foot bitten; several days afterwards in play with some other children, he snapped at one of them. The cry of "gone mad" was immediately raised. For the next seven days he had from ten to twenty convulsions a day, and was tied or held most of the time; he was brought to me the 14th day after the bite and the seventh after the first convulsion. The wound on the foot had healed and his general appearance was good. As soon as he got into the office a seizure began, and lasted about thirty minutes. There were contractions of all the limbs, but they were neither constant nor rythmical; only the whites of the eyes were visible. The facial expression was sardonic; the mouth was full of froth; there was apparent loss of consciousness; still he bit and snapped at everything in his reach. Not allowing him to be held, I watched him through the entire seizure, and will say that it was anything but a pleasant sight. I prescribed fair doses of a bromide and an anthelmintic, with orders to use no force to restrain him, telling him that he was no more mad than I, and would soon be well. In three days his father returned, reporting that the day after leaving my office he had passed a large lumbricoid worm, and had only had a few slight convulsions since, all of these at the sight of a certain playmate. Bromides were continued, and he was told that the next convulsion would be the signal for a first-class whipping, which dose has never been required.

From close observation and considerable interest in this case, it is my honest opinion that had he remained in his first surroundings the boy would have died. It is scarce worth while to go into an analysis of this case; the combined causes of the condition are too obvious.

During the late extended discussion of rabies in conjunction with the treatment of Pasteur, a book by Edward Mayhew, M. R. C. V. S., has been often quoted as the only scientific work on the dog. He says as follows, speaking of fits in the dog: "It is standing still as if stupefied, suddenly it emits a strange, loud, guttural sound, and then falls upon its side, continuing to cry, but more feebly and more naturally. It will bite any one who, during the existence of the attack, incautiously attempts to lay hold of it; its limbs, at first stretched rigidly out, are alternately, with returning volition, put into violent motion; the eye is protruded and foam covers the mouth. When the convulsion has subsided, the dog raises his head and stares about; after which it would, if left alone, start at its utmost pace and run heaven only knows where. Should idle men and foolish boys behold a dog wildly run onward after having come out of a fit, and raise the cry of "mad dog," the fate of the poor animal is then sealed, as fear is devoid of discrimination or pity. Half the dogs killed as rabid are those in this condition, scampering under the impulse of returning sensation. That is the popular description of a mad dog, and one of the most common diseases of the animal. I suppose I have seen twenty cases of it. The following is his description of rabies:

"The dog that is going mad feels unwell for a long time prior to the development of the disease. He is ill but he does not know what ails him. He feels nasty, dissatisfied with everything, vexed without a reason; and greatly against his better nature, very snappish. Feeling thus, he longs to avoid all annoyance by being alone. This sensation induces him to seek solitude; but there is another reason which decides his choice of a resting place. The light inflicts upon him intense agony; the sun is to him an instrument of torture which he therefore studies to avoid. So anxious is he for liquids and so depraved are his appetites that no sooner has he passed a little urine that he turns around to lick it up. He is now altogether changed, still he does not desire to bite mankind, he rather endeavors to avoid society, he takes long journeys of thirty or forty miles in extent, and lengthened by all kinds of accidents to vent his restless desire for motion. When on these journeys he does not walk, that would be too formal and measured a pace for an animal whose whole frame quivers with excitement. He does not run, that would be too great an exertion for an animal whose body is the abode of a deadly sickness. He proceeds in a slouching manner, in a kind of trot, a movement neither run nor walk, and his aspect is dejected; *his eyes do not glare and stare but they are dull and retracted.* His appearance is very characteristic, and if once seen can never afterwards be mistaken. In this state he will travel the most dusty roads, his tongue hanging *dry* from his open mouth, from which, however, *there drops no foam.* His course is not straight, how could it be, since at this period it is doubtful if he sees at all. His desire is to journey unnoticed. If no one notices him he gladly passes by them. He is very ill, *he cannot stay to bite.* If nevertheless anything oppose his progress he will, as if by impulse, snap, as a man in a similar state might strike."

Did any of my hearers ever think of a mad dog in that light ?

In regard to frequency in speaking of some experiments made to induce rabies, he says as follows:

"It is rarely that more than one mad dog appears at a time in England; so, to perfect their experiment it would be requisite for the French philosophers to procure all the specimens of the canine species in this island and doom them to torture."

One of the largest dealers in dogs in this country, who has been in the business half a century, told me sometime since that he had never seen a rabid dog, and at that time he had about a thousand dogs on hand. So the disease cannot be so very frequent in the animal. When it does occur, according to all authorities, the season appears to have no influence on its frequency. Dr. Hermann M. Biggs, in his valuable article on the etiology of rabies and the method of M. Pasteur for its prevention, says as follows:

"It is a matter of the greatest importance that those fallacies should be corrected which are so prevalent among the laity, and to no small extent among the profession, in regard to the influence of climate, season, hunger, thirst, food, pain, anger, an unsatisfied sexual desire, and the thousand other causes that have at one time or another been adduced as predisposing to, or producing the disease in dogs. There are no better nor more reasonable grounds for believing that any one or all of these influences combined can bring about the development of a single case of rabies, than that unfavorable sanitary conditions can produce small-pox when the specific *contagium vivium* of the disease is not present. So strong has been the popular belief in the effects of the season in the production of rabies, that not even the testimony of indisputable facts has been patent enough to dispel it.

It has been almost a universal belief since the earliest times that the canine species are more susceptible and liable to an attack of this malady during the hot weather than at other seasons of the year, and for a certain period during the summer and autumn months many precautions have been taken to protect man against the possible dangers arising from this cause. During this period, we find that in many cities there are municipal ordinances requiring that all dogs at large in the streets shall be provided with muzzles, while during the remainder of the year they are left free. The danger from the possible development of the disease has been supposed to be proportionate to the intensity of the heat. This supposition has been maintained in spite of the fact that it has been shown in the most unmistakable manner, by large masses of statistics collected with the greatest care in France and Germany, that the disease is not more prevalent in the summer months than at other seasons of the year, and that, if there is any preponderance of cases in any season, it is rather in the winter and spring than in the summer. Indeed Dr. Roll, of the Vienna Veterinary Institute asserts that the disease is more frequent in mild than in hot summers."

Is the disease as frequent in man as we are led to believe? Did any of my hearers ever hear of a professional dog catcher having the disease? They certainly get bit often enough. The same could be asked in regard to dog dealers and sportsmen.

During the five years that I was fairly conversant with the hospital news of New York City, I never heard of a case in any of the institutions. Dr. Carleton, of Conn., who enjoyed the largest private surgical practice of any man that I ever knew, extending over a period of thirty years, never saw a case.

Dr. W. T. Lusk, who has had many years hospital experience, writes me that he has never had or seen a case.

Dr. W. D. Mitchell, of St. Vincents, writes: "I have never seen a case, neither has there been any treated in this hospital". St. Vincents was established in 1849, and last year had 2118 cases, of which 1708 were ambulance calls; also 682 out-door patients were treated. Dr. W. N. Belcher, who has been connected with hospital work in Brooklyn for six years, and is at present assistant in the Hoagland Laboratory, writes: "I can find no record of any case being treated in the Long Island Hospital. In the death record of the board of health for 1884-5-6-7 and 8, I find only one death reported; that was in 1886. So far as I can learn, Dr. Skenes experience with hydrophobia has been *nil*.

Dr. Wm. B. Anderson, who has been connected with Bellevue Hospital since 1879, writes: "I saw a positive case in the spring of 1881 in Bellevue Hospital." My recollection is that Bellevue Hospital treats in the two departments considerable over 20,000 cases a year.

You will allow that the combined experience above covers several hundreds of thousands of sick people. Consequently by a rational process of reasoning, we would call it a very rare disease.

Still our morning paper, in our supposed hydrophobia season, usually gives us the full details of a case.

Do not infer that I deny the existence of the disease any more than I do that of glanders, or anthymycosis. I do think that it is a crying shame that popular opinion is such that the majority of our women and children cannot see a dog without associating it with hydrophobia, and when a person, not used to dogs, gets bitten, the fear of hydrophobia hangs on him for years, when his chances are not one in thousands of contracting the disease. The dog is of course invariably killed before a diagnosis can be made.

In conclusion I wish to remark that the dread of this disease has caused much more suffering to human beings than the disease itself.

